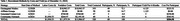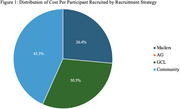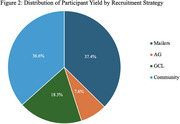# Evaluating Preliminary Recruitment Strategies for Research Studies in Older Women at Higher Risk of AD

**DOI:** 10.1002/alz70860_103930

**Published:** 2025-12-23

**Authors:** Ella T Lifset, Manjot Kaur, Kayla Ponce, Melanie A Dratva, Nadine C. Heyworth, Erin E. Sundermann, Sarah J Banks

**Affiliations:** ^1^ University of California, San Diego, La Jolla, CA, USA; ^2^ University of California, Los Angeles, Los Angeles, CA, USA

## Abstract

**Background:**

Recruitment for Alzheimer's disease (AD) research is challenging, with many factors impacting screening and enrollment. Data on effectiveness and relative cost of recruitment strategies are rarely shared, yet comprise an important aspect of research planning. We evaluated preliminary recruitment data in an ongoing, two‐year, longitudinal study of older women at higher risk for AD. The study involves PET and MRI neuroimaging, lumbar puncture, cognitive testing, and wearable health monitors. Costs per participant and enrollment yield were compared across recruitment strategies.

**Method:**

The Women: Inflammation and Tau Study (WITS) has contacted 540 interested individuals between 12/2020‐12/2024. Major eligibility requirements are: ≥65 years, female, no dementia diagnosis, mild impairment (T‐MoCA score 13‐20), and AD polygenic hazard score >50th percentile. Recruitment strategies were sorted into four categories: Geotargeted Mailers, Affiliate Group referrals (AGs; from collaborator studies), Generated Contact Lists (GCLs; from electronic health records and research registries), and Community outreach (e.g., education talks, local news segment) with Mailers and AGs utilized for 4 years, and GCL and Community‐based methods introduced 6 months ago.

**Result:**

Of 540 individuals contacted, 213 completed screening, and 70 were enrolled. Cost per participant varied, with AGs being most cost‐effective (no cost), followed by Mailers ($223), GCLs ($256), and Community outreach ($365.92) as most‐expensive (Table 1). The three cost‐associated strategies comprised 26.4%, 30.3%, and 43.3% of the recruitment budget, respectively (Figure 1). Across a six‐month interval, Mailers yielded the highest average number of participants enrolled (*n* = 6.12, 37.4%), followed closely by Community outreach (*n* = 6.00, 36.6%). GCLs (*n* = 3.00, 18.3%) and AGs (*n* = 1.12, 7.6%) yielded fewer average participants enrolled (Table 1, Figure 2).

**Conclusion:**

This preliminary work provides insight into the effectiveness of WITS recruitment strategies, and emphasizes needs for reserving significant financial investment for recruitment (∼$200‐$350 per participant). Although community outreach was the most‐expensive strategy, it was one of the most‐effective, justifying sustained investment into community‐based efforts and further informing best practices for recruitment across AD research. Notably, the current WITS participant cohort lacks diversity, possibly due to early geotargeted recruitment methods, but novel strategies to engage underrepresented groups are being implemented and will be detailed in the poster session.